# Diagnostic Value of Nuclear Hybrid Imaging in Malignant Struma Ovarii: A Systematic Review of Case Reports

**DOI:** 10.3390/diagnostics14232630

**Published:** 2024-11-22

**Authors:** Claudiu Peștean, Doina Piciu

**Affiliations:** 1 Faculty of Medicine, Department of Medical Imaging and Nuclear Medicine, “Iuliu Hațieganu” University of Medicine and Pharmacy, 8 V. Babeș St., 400006 Cluj-Napoca, Romania; doina.piciu@gmail.com; 2 “Ion Chiricuță” Oncology Institute, 34-36 Republicii st., 400015 Cluj-Napoca, Romania; 3 Affidea CT Clinic, 65-67 Republicii St., 400489 Cluj-Napoca, Romania

**Keywords:** SPECT/CT, PET/CT, PET/MRI, malignant struma ovarii, hybrid imaging

## Abstract

Background: Struma ovarii is a rare tumor, a type of ovarian mature teratoma consisting over 50% of its mass in thyroid ectopic tissue; 5% to 10% of cases, as described in the literature, are malignant and well known as malignant struma ovarii or thyroid cancer from struma ovarii. Due to the limited number of malignant struma ovarii cases, the diagnostic and therapeutic approach of malignant struma ovarii lacks in standardization. Methods: We performed a comprehensive search on the English language PubMed and Google Scholar. We used specific controlled keywords “PET CT”, “SPECT CT”, “PET MRI”, “malignant struma ovarii”, “hybrid imaging” and “mature ovarian teratoma”. Upon the retrieval of potential articles, we analyzed them for their eligibility. The inclusion criteria were: articles discussing the role of PET/CT and SPECT/CT hybrid imaging in malignant struma ovarii, full-text articles on the topic of interest and English publications. The exclusion criteria were articles not directly related to the hybrid imaging and not discussing the subject of malignant struma ovarii. Results: A total of 64 articles were screened, 35 duplicates were eliminated, 15 articles excluded and a total number of 14 articles were included for this systematic review, 13 of them being case reports and one being a case report with a systematic review. F-18 FDG PET/CT contributed in seven cases (50%), I-131 NaI SPECT/CT in seven cases (50%) and I-124 NaI PET/CT in two cases (14.29%). In two cases, 131 NaI SPECT/CT and F-18 FDG PET/CT were used as complementary investigation tools. The hybrid imaging methods used as a part of the diagnostic strategy were accompanied by several diagnostic alternatives: ultrasounds, CT, MRI, I-131 NaI WBS and I-123 NaI WBS. Conclusions: There is no consistent or standardized diagnostic and therapeutic approach for malignant struma ovarii. Hybrid imaging methods may be of great value in initial diagnostic and the association of F-18 FDG PET/CT and I-131 NaI SPECT/CT is a successful diagnostic approach. The association of hybrid imaging with other diagnostic imaging alternatives in initial diagnostic and follow up is essential.

## 1. Introduction

Struma ovarii is a rare tumor, a type of ovarian mature teratoma with over 50% of its mass being thyroid ectopic tissue; it was reported for the first time in 1889 [1]. It accounts for 3% of mature ovarian teratoma and 1% of all ovarian tumors [2]. Benign histological types represent most cases, but 5% to 10% of cases, as described in the literature, are malignant and are well known as malignant struma ovarii or thyroid cancer from struma ovarii [3,4]. There are no significant difference between the benign and malignant struma ovarii related to the age of the patient at the moment of diagnosis [4]. Malignant struma ovarii occur more often in the left ovary as a unilateral adnexal mass [5].

Having its extremely rare occurrence, there are sporadic articles in the literature based on malignant struma ovarii and there is a lack of consistent studies, most of them being case reports or short series. The diagnostic and also the therapeutic approach of malignant struma ovarii lack in standardization and, sometimes, the rareness of this disease may lead to the inability in providing the most suitable treatment.

### 1.1. Histological Considerations in Malignant Struma Ovarii

The histopathological classification of malignant struma ovarii relays a similar approach to that of thyroid carcinoma [6]. The most prevalent type is the papillary thyroid carcinoma, followed by follicular thyroid carcinoma, follicular variant of papillary thyroid carcinoma and highly differentiated follicular carcinoma [2,4]. The less prevalent is poorly differentiated thyroid carcinoma and medullary thyroid carcinoma [1,4].

The malignant struma ovarii may be associated with thyroid dysfunctions like Grave’s disease or hypothyroidism, Hashimoto’s thyroiditis, thyroid nodules, but also, there are cases in the literature that describe the association of malignant struma ovarii with thyroid cancer [1,4]. The studies reporting on molecular evaluation of malignant struma ovarii mentioned the presence of gene mutations in RAS (rat sarcoma), BRAF (B-rapidly accelerated fibrosarcoma), and KIT (receptor tyrosine kinase) proto-oncogenes, and also, mutations in RAS, BRAF and TERT (telomerase reverse transcriptase) promoters [4]. These genetic findings are the proof that malignant struma ovarii has the same molecular mechanism like the thyroid carcinomas [4].

### 1.2. Disease Management Considerations in Malignant Struma Ovarii

Malignant struma ovarii has a controversial therapeutic approach, since no large cohorts of patients are available to be able to establish effective risk and prognostic factors, survival and recurrence rates. The main treatment remains the surgical approach. The extension of the procedure relies on the extent of the disease and fertility requirements and sometimes the contralateral ovary preservation is necessary [2,4].

The adjuvant therapies are largely disputed, but some authors propose the prophylactic thyroidectomy and radioiodine therapy for certain categories of patients with high risk of recurrence. For this, a proper risk stratification is helpful, and tumors larger than 2 cm, with extra-ovarian extension and aggressive histological features might be risk factors leading to the decision to perform thyroidectomy and radioiodine therapy [1,4,7]. For a better response to radioiodine therapy, the administration prior to treatment of recombinant human thyrotropin will boost the absorption of radioiodine I-131 increasing the NIS (natrium-iodine symporters) activity. Chemotherapy in advanced ovarian tumors or recurrence and metastatic disease may be applied but with poor outcomes [2].

The patient monitoring will consist of long-term follow-up, at least 10 years, with close clinical surveillance, periodic serum Thyroglobulin and serum Thyroglobulin-antibodies dynamic measurements and also with whole-body scans [3].

### 1.3. Diagnostic Imaging Considerations in Struma Ovarii

The histological features that are post-surgically found usually diagnose most of the cases of malignant struma ovarii, although the symptoms and signs like abdominal/pelvic masses or pain, irregular menstruation or ascites are reasons to further investigate the patients that usually undergo gynecological evaluation. The endocrinologists usually encounter the case only after the gynecologist’s referral, which is often after the initial surgical treatment [4].

The imaging tool of first choice is usually abdominal ultrasound that might reveal heterogenic masses with echogenic solid and transonic cystic parts, abundant blood flow signal on Doppler imaging, having no specific imaging aspects [8].

CT (computer tomography) imaging corresponds to the macroscopic aspect of malignant struma ovarii, showing multiloculated masses with different densities, with solid components, cystic parts and even fat and calcifications [8].

MRI (magnetic resonance imaging) depicts the intratumoral fat as high signal intensity on T1-weighted images and the mucinous colloid as low signal on T1- and T2-weighted images [8].

#### 1.3.1. SPECT/CT Hybrid Imaging

SPECT/CT (single photon emission tomography combined with computer tomography) hybrid imaging may be of great value because it exploits and combines two different diagnostic principles.

The CT features of malignant struma ovarii are accorded to the tissue densities of the tumor and the CT findings may vary as already described. SPECT has the ability to use molecules as probes that highlights a certain metabolic pathway. Struma ovarii is a rare uncommon disease and malignant tissue of the cancers arising in struma ovarii tend to be very well differentiated [9],

Radioiodine I-131 sodium iodide (I-131 NaI) is a suitable radioisotope for diagnostic and therapeutic procedures in malignant thyroid disease. It is a dual isotope and a gamma and beta emitter; it has an abundance of 81.5% in gamma photons with an energy of 364 keV and a half-life of 8,02 days, useful in diagnostic procedures [10,11]. Special attention is given to diagnostic procedures with I-131 in all differentiated thyroid malignant tissues arising from orthotropic thyroid or ectopic tissue; even malignant struma ovarii and precautions are considered related to the stunning effect that a diagnostic dose may produce to the thyroid tissue which may impair the following radioiodine therapy.

Radioiodine I-123 sodium iodine (I-131 NaI) has the same pharmacokinetic way, it is the most preferred radiopharmaceutical for thyroid imaging since it is a pure gamma emitter isotope with the most predominant photon energy at 127 and 159 keV and a half-life of 13.22 h. Having these properties, it does not produce unnecessary radiation burden caused by any beta emission and it does not produce a stunning effect due to its shorter half-life [10].

When malignant struma ovarii is suspected or diagnosed, the need for SPECT/CT imaging may be considered in those cases where total thyroidectomy was undertaken, but special care should be taken related to the stunning effect of I-131 if it is the radioisotope of choice. In order to avoid that, small amounts or I-131 NaI should be used, typically 37–111 MBq. The preferable choice is I-123 NaI to avoid all the therapeutic impairments. The post-therapeutic I-131 SPECT/CT is of great value since it may reveal morphological information of equivocal lesions, having good specificity and higher sensitivity [10]. The combination between SPECT and CT will better asses the size, location and the avidity for iodine and thus, will better guide the clinicians for further disease management decisions [12].

#### 1.3.2. PET/CT Hybrid Imaging

PET/CT (positron emission tomography combined with computed tomography) is a hybrid imaging method that has already proven its major advantage in oncological pathologies, being a key diagnostic tool that may provide essential diagnostic information data in initial diagnostic, staging/extension of disease, risk stratification and treatment response evaluation [13].

F-18 is the most widely used radioisotope in PET. It is a positron emitter with a long enough physical half-life of 110 min, able to produce 511 keV of annihilation photons suitable to investigate, in a convenient way, different pathologies.

F-18 FDG remains the first choice from the available PET radiopharmaceuticals panel on the market. F-18 FDG is very suitable for assessing the exacerbated metabolic activities and an excellent radiopharmaceutical method for cancer detection, staging, disease recurrence and treatment response evaluation [14]. Due the fact that malignant struma ovarii is represented by thyroid tissue, the F-18 FDG PET/CT may be used as a diagnostic tool to evaluate its metabolic characteristics, as for thyroid cancer, especially in high risk patients [10]. F-18 FDG PET/CT may be useful in those cases when de-differentiation occurs and the metabolic cellular activity increases. Less prevalent, the poorly differentiated thyroid carcinoma, or medullary thyroid carcinoma types, that are more aggressive than the well-differentiated types, may be candidates for F-18 FDG PET/CT examination [10,13,15,16].

In medullary thyroid carcinoma (MTC), F-18 DOPA PET/CT might be the most specific molecular-imaging tool [10,15]. Thus, in malignant struma ovarii containing MTC, this specific imaging tool may add diagnostic value. In the same light, the possibility to detect osseous metastases and extra-osseous metastases with calcifications from MTC using F-18 NaF PET/CT [10,15] should be mentioned.

It should also be mentioned that medullary thyroid carcinoma cells may express somatostatin receptors; Ga-68 DOTATATE PET/CT, as an imaging method that can detect medullary thyroid carcinoma that express somatostatin receptors, can exploit this particularity [10,15].

I-124 follows the same pharmacokinetics as other radioiodine radiopharmaceuticals previously discussed: the ability of well-differentiated thyroid malignant cell to transport the iodine and to use it in thyroid hormone production. With a half-life of 4.2 days and its positron emissions, I-124 in the form of I-124 NaI is successfully used as radiopharmaceutical in PET/CT, a technique that offers better spatial resolution, better specificity and no stunning effect [15].

Recently, a novel PET radiopharmaceutical procedure has been successfully used in many oncological pathologies targeting the cancer-associated fibroblasts that are the most present cells in tumor stroma. These cells are associated with high expression of the fibroblast-associated protein (FAP), normally very limited in sane tissues. The radiopharmaceutical therapy in discussion is Ga-68- or F-18-radiolabelled FAP inhibitors (FAPI) [17,18].

This systematic review aimed to reveal the role that hybrid imaging tools like PET/CT and SPECT/CT can play in malignant struma ovarii diagnosis. Another declared aim was to find their predictive value and how these imaging methods can be used for diagnosis and follow-up.

## 2. Materials and Methods

For this systematic review we performed a search on the PubMed and Google Scholar international databases. We used specific controlled keywords including “PET CT”, “SPECT CT”, “PET MRI”, “malignant struma ovarii”, “hybrid imaging” and “mature ovarian teratoma”. The keywords we used for the search were included in the following syntaxes: “SPECT CT in malignant struma ovarii”, “PET CT in malignant struma ovarii”, “PET MRI in malignant struma ovarii” “hybrid imaging in malignant struma ovarii”, “SPECT CT in mature ovarian teratoma”, “PET CT in mature ovarian teratoma”, “PET MRI in mature ovarian teratoma” and “hybrid imaging in mature teratoma” in order to have a comprehensive selection discussing the topic of hybrid imaging in malignant struma ovarii. However, no articles discussing PET/MRI were found.

The selection and the analysis of the articles were performed in September 2024 and October 2024 and we included articles from the earliest record to October 2024.

The inclusion criteria were: articles discussing the role of PET/CT, PET/MRI and SPECT/CT hybrid imaging tools in malignant struma ovarii, full-text articles and English publications on the topic of interest.

The exclusion criteria included articles not specifically connected to hybrid imaging and not discussing the subject of malignant struma ovarii.

Upon the retrieval of potential articles, we analyzed them for their eligibility. The studies were analyzed together by two reviewers. A total number of 62 articles were identified from the databases search and two more articles were added from the reference lists of potential articles. We then identified the duplicate articles and removed 35 duplicates. We further refined the selection, and we excluded the articles consisting of non-full text articles, and others matching the exclusion criteria. A total number of 11 articles were excluded. By reviewing the content of the articles, we identified one article presenting misleading and incorrect data showing a radioiodine whole body scan presented as Tc-99m pertechnetate thyroid scintigraphy, and the article was excluded.

**Figure 1:**
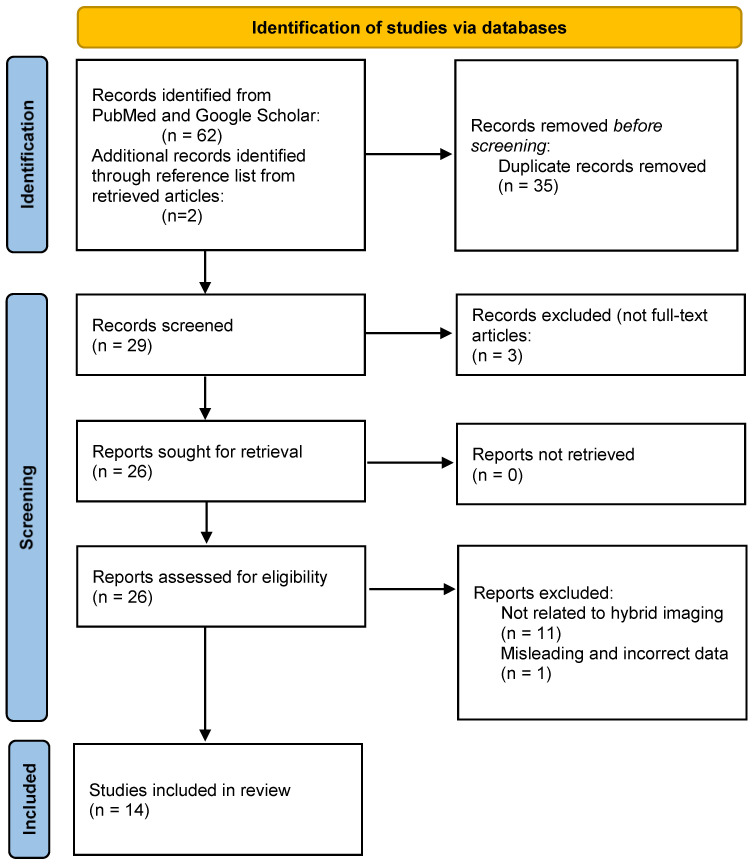
Selection criteria (PRISMA flow diagram).

After we applied the inclusion and exclusion criteria, a total number of 14 articles were considered eligible for this systematic review, as it is represented in Figure 1.

We used Microsoft Excel 2021 for data collection, table data organization and graphic and chart generation.

We collected information about the year and origin country of the report, type of hybrid imaging method used, additional medical imaging, age of the patients, histological data, localization of the tumor, the presence of the metastases, association with thyroidectomy, involvement of radioiodine therapy, association with other therapies and surgical approach.

## 3. Results

The eligible articles included in this systematic review consist of thirteen case reports and one case report with a literature review arguing the role of hybrid imaging SPECT/CT and PET/CT in malignant struma ovarii. The studies were published between 2005 and 2023. The articles analyzed in the present systematic review are listed in Table 1.

After we analyzed the age of the patients included in the present systematic review, we found a median value of 44.5 with ages ranging from 11 to 55 years old. The particularities of the case reports are listed in Table 2.

**Figure 2:**
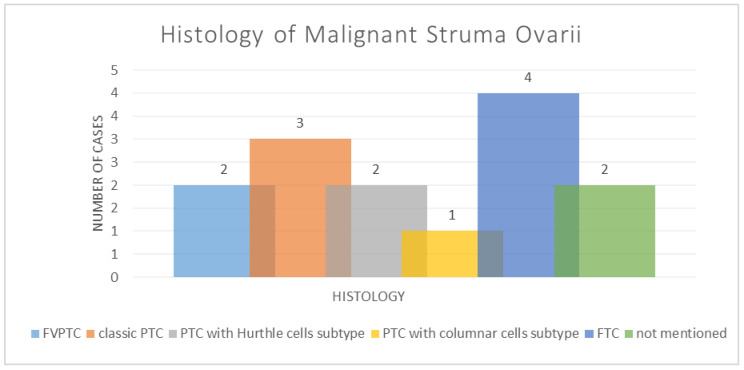
The histology of malignant struma ovarii (FVPTC—follicular variant of papillary thyroid carcinoma; PTC—papillary thyroid carcinoma; FTC—follicular thyroid carcinoma).

From these fourteen cases of malignant struma ovarii, according to their histology, two were cases of follicular variant of papillary thyroid carcinoma (14.29%), three cases of classic papillary thyroid carcinoma (21.43%), two cases of papillary thyroid carcinoma of Hurthle cells subtype (14.29%), one case of papillary thyroid carcinoma with columnar cells subtype (7.14%) and four cases of follicular thyroid carcinoma (28.57%). However, two cases had unmentioned histology in the content of studies (14.29%); see Figure 2. A total of seven cases described malignant struma ovarii localized in the right ovary (50%), five cases in the left ovary (42.86%) and two cases were presented without this detail (7.14%).

**Figure 3:**
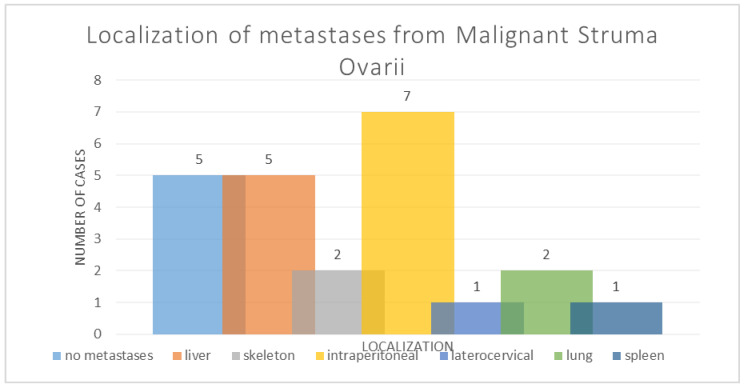
The localization metastases from malignant struma ovarii.

From all fourteen cases, five cases had no metastases described (37.51%, and 64.29% of cases being with metastatic disease). As for the localization of the metastatic foci, seven cases presented intraperitoneal metastases (50%), five cases described liver metastases (37.51%), two cases had different skeletal localizations (14.29), one case presented latero-cervical metastases (7.14%), and one case also described spleen metastases (7.14%); see Figure 3.

Just one case of malignant struma ovarii was associated with orthotropic thyroid carcinoma and ten cases benefitted from radioiodine metabolic therapy. All cases that were treated with radioiodine therapy were cases that previously underwent total thyroidectomy. Two cases had associated adjuvant therapies like systemic chemotherapy and retinoid acid therapy.

**Figure 4:**
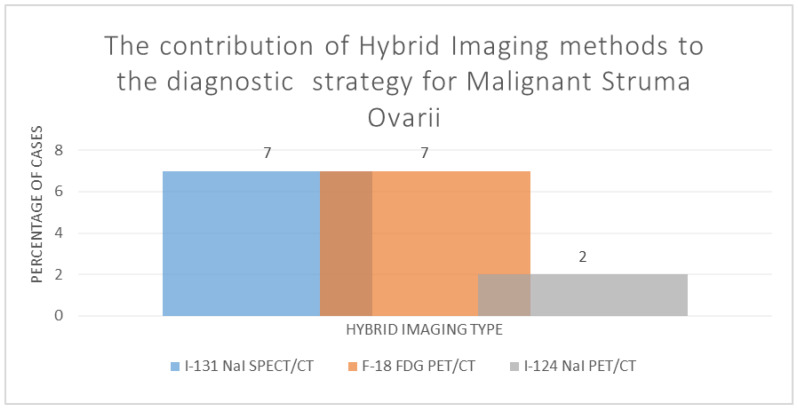
The contribution of hybrid imaging methods to the diagnostic strategy for malignant struma ovarii (I-131 NaI SPECT/CT—I-131 (natrium iodine) radioiodine single photon emission tomography fused with computer tomography; F-18 FDG PET/CT F-18 fluorine fluoro-deoxi-glucose positron emission tomography fused with computer tomography; I-124 NaI PET/CT—I-124 (natrium iodine) radioiodine positron emission tomography fused with computer tomography).

Related to the hybrid imaging methods used in cases described in the reports included as eligible for the present systematic review, F-18 FDG PET/CT contributed to seven cases (50%), I-131 NaI SPECT/CT to seven cases (50%) and I-124 NaI PET/CT to two cases (14.29%). However, no cases were identified among the report cases that mentioned the use of I-123 NaI SPECT/CT. There were no cases where PET/MRI was used as an investigation tool; see Figure 4. In two cases, 131 NaI SPECT/CT and F-18 FDG PET/CT were used as complementary investigation tools.

All cases investigated via radioiodine-based imaging methods were cases associated with total thyroidectomy. Except one case, all of these cases were treated with radioiodine therapy.

**Figure 5:**
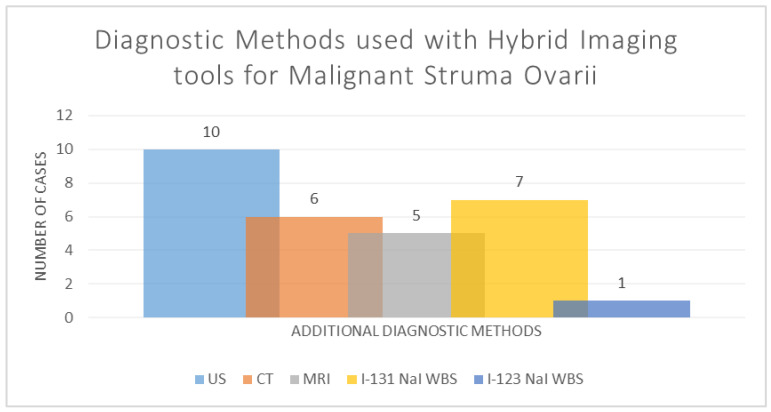
The diagnostic methods used with hybrid imaging tools for malignant struma ovarii (I-131 NaI WBS—I-131 (natrium iodine) radioiodine whole body scan; CT—computer tomography; US—ultrasound; MRI—magnetic resonance imaging; I-123 NaI WBS—I-123 (natrium iodine) radioiodine whole body scan).

Moreover, the hybrid imaging methods used as a part of the diagnostic strategy were accompanied by several diagnostic alternatives: ultrasounds in ten cases (71.43%), CT in six cases (42.85%), MRI in five cases (35.71%), I-131 NaI WBS in seven cases (50%) and I-123 NaI WBS in one case (7.14); see Figure 5.

## 4. Discussion

Malignant struma ovarii is a rare condition with an incidence lower than 5% of all ovarian teratoma [5]. It is often described with a clinical presentation that suggests the presence of metastatic disease. This is demonstrated via objective clinical exams and via medical imaging tools. In our systematic review, the presence of metastatic disease was described in 64.29% of cases. Seifert et al. mention that the bone metastases arising from differentiated thyroid tissue are often radioiodine-refractory, therefore, relatively higher activities of I-131 (11 GBq) should be considered for radioiodine therapy, however there are no recommendations in respect to this [29].

When no radioiodine uptake is demonstrated for the metastases that arise from malignant struma ovarii with differentiated histology, Wu et al. suggest that the association of radioiodine therapy with TK (tyrosine kinase) inhibitor therapy could be a successful alternative as they presented for liver and spleen metastases without radioiodine uptake [26].

Since it is not recognized and there is no standardization for treatment and diagnosis in malignant struma ovarii, many diagnostic and disease management principles are the same as for differentiated thyroid carcinoma arising from orthotropic thyroid tissue. In this light, Wu et al. suggest that rTSH (recombinant human thyrotropin) may be helpful to increase the radioiodine uptake in cases when it is difficult to raise the endogenous TSH [26]. The use of rTSH is also mentioned by Gobitti et al. to avoid the progression of disease in some cases, due to the hormonal withdrawal that is necessary for the patient preparation in adjuvant radioiodine therapy [5].

Thyroglobulin represents an efficient tumoral marker for monitoring and recurrence detection in malignant struma ovarii with differentiated histology as Seifert et al. demonstrate [29]. When its values also remain elevated after adjuvant thyroidectomy, it may suggest metastatic persistence of malignant struma ovarii, if we consider the conclusions of Middelbeek et al. [25].

In cases where the dedifferentiation of thyroid malignant tissue in the malignant struma ovarii occurs, it may be useful to partner radioiodine therapy with retinoid acid therapy, the latter being a solution for the redifferentiation of thyroid malignant tissue which will optimize the subsequent radioiodine treatment, as Seo et al. propose [23].

The F-18 FDG PET/CT is reflecting the disease biology and thus, the behavior, since it is based on the metabolic rate of malignant tissue. Considering this, Ranade et al. conclude that in malignant struma ovarii lesions, F-18 FDG PETR/CT usually depicts low avidity of the lesions arisen from malignant struma ovarii, a situation that might be explained by the presence of differentiated thyroid tissue which is known to have a mild behavior [24].

Yamauchi et al. affirmed that F-18 FDG PET/CT has a mild uptake in malignant struma ovarii primary lesions since the tumor itself has a mixed component of malignant thyroid tissue [3], thus sometimes, F-18 FDG PET/CT has unspecific aspects, and even no uptake, as Wolff et al. declare [20].

Seo et al. found liver lesions with unspecific uptake on F-18 FDG PET/CT, probably due to their metabolic heterogeneity [23].

On the other hand, Gobitti et al. concluded that F-18 FDG PET/CT was useful for restaging [5], and Fujiwara found F-18 FDG PET/CT of value in depicting distant metastases and recurrence, often associated with high aggressivity [27]. This affirmation is sustained also by the study of Al Shammaa et al. which concluded that F-18 FDg PET/CT may be used in assessing the disease progression and recurrence [30].

Fujiwara et al. also affirmed that F-18 FDG ET/CT may give a differentiated diagnostic between malignant struma ovarii and the benign form, since the latter does not present any uptake for F-18 FDG [27].

Cherng et al. affirmed that I-131 NaI SPECT/CT has an important role in confirming the origin of radioiodine avid lesions to the ovarian structure in cases of suspicion of malignant struma ovarii [19].

Lager et al. found in their study that I-131 NaI SPECT/CT may be a good diagnostic tool. The main advantage is that that I-131 NaI SPECT/CT is able to detect lesions missed on planar I-131 WBS [28].

Koo et al. affirmed that the combination between I-131 NaI SPECT/CT and F-18 FDG PET/CT may be a successful preoperative approach in malignant struma ovarii [21]. This is a successful combination since I-131 NaI SPECT/CT may detect the I-131 avid lesions as Seo et al. affirmed [23], and F-18 FDG PET/CT is useful for the evaluation of distant disease according to Fujiwara et al. [27].

It is obvious that in cases of malignant struma ovarii with differentiated histology, the radioiodine-based imaging tools are of great importance due to their specificity, but the best option seems to be I-124 NaI PET/CT, due to its superior technical advantages in terms of sensitivity and resolution, an aspect underlined by Seifert et al. in their report [29]. Another advantage is the possibility to avoid the stunning effect.

The present review has some limitations, mainly because the low number of available reports, and the lack of large cohort studies aimed at the topic of malignant struma ovarii and hybrid imaging alternatives. Another limitation is the inexistence of cases reported about the PET/MRI hybrid imaging tool.

Due to the limited data available in the literature, it is difficult to have a standardization for diagnostic approach in malignant struma ovarii. This review might be an insight of the perspective offered by the nuclear hybrid imaging in malignant struma ovarii, but further research would be useful, especially if more cases are reported and the panel of hybrid nuclear medicine alternatives extends (e.g., PET/MRI, PET/CT using other radiopharmaceuticals).

## 5. Conclusions

Malignant struma ovarii is a rare disease and unsystematized. Peculiar data can be taken from the articles available in international databases, with articles mainly consisting of case reports or short series. There is no consistent or standardized diagnostic and therapeutic approach for malignant struma ovarii.

Hybrid imaging methods may be of great importance in initial diagnostics due to their ability to assess the lesions using a double functional and morphological perspective. This hallmark is the main advantage against the other stand-alone morphological imaging tools that are unable to functionally asses the lesions. The association of F-18 FDG PET/CT and I-131 NaI SPECT/CT is a successful diagnostic approach in malignant struma ovarii. In differentiated histological types of malignant struma ovarii, I-124 NaI PET/CT appears to be superior to I-131 NaI SPECT/CT as a diagnostic method. The association of hybrid imaging with other diagnostic imaging alternatives in initial diagnostics and follow up of malignant struma ovarii is essential. The diagnostic, follow-up and therapeutic approach to malignant struma ovarii with differentiated histology may have the same principles as that of orthotropic differentiated thyroid cancer.

## Figures and Tables

**Table 1:** The articles analyzed in the review.

NrCrt	Author	Year	Country	Hybrid Imaging	Additional Imaging
1	Cherng et al. [19]	2005	Taiwan	I-131 NaI SPECT/CT	I-131 NaI WBS, CT, US
2	Wolff et al. [20]	2010	USA	F-18 FDG PET/CT	I-131 NaI WBS, CT, MRI
3	Koo et al. [21]	2012	USA	I-131 NaI SPECT/CT	I-131 NaI WBS
4	Lopci et al. [22]	2013	Italy	I-124 NaI PET/CT	no
5	Seo et al. [23]	2014	Korea	I-131 NaI SPECT/CT, F-18 FDG PET/CT	MRI, US
6	Ranade et al. [24]	2015	India	F-18 FDG PET/CT	I-131 NaI WBS, CT, US
7	Middelbeek et al. [25]	2017	USA	I-131 NaI SPECT/CT	I-123 NaI WBS, US
8	Gobitti et al. [5]	2017	Italy	F-18 FDG PET/CT, I-131 NaI SPECT/CT	CT, US
9	Wu et al. [26]	2018	China	I-131 NaI SPECT/CT	I-131 NaI WBS, US
10	Fujiwara et al. * [27]	2018	Japan	F-18 FDG PET/CT	CT, MRI, US
11	Lager et al. [28]	2018	USA	I-131 NaI SPECT/CT	I-131 NaI WBS
12	Seifert et al. [29]	2019	Germany	I-124 NaI PET/CT	I-131 NaI WBS, US
13	Yamauki et al. [3]	2022	Japan	F-18 FDG PET/CT	CT, MRI, US
14	Al-Shammaa et al. [30]	2023	Jordan	F-18 FDG PET/CT	MRI, US

* case study with literature report; I-131 NaI SPECT/CT—I-131 (natrium iodine) radioiodine single photon emission tomography fused with computer tomography; I-131 NaI WBS—I-131 (natrium iodine) radioiodine whole body scan; CT—computer tomography; US—ultrasound; MRI—magnetic resonance imaging; F-18 FDG PET/CT—F-18 fluorine fluoro-deoxi-glucose positron emission tomography fused with computer tomography; I-124 NaI PET/CT—I-124 (natrium iodine) radioiodine positron emission tomography fused with computer tomography; I-123 NaI WBS—I-123 (natrium iodine) radioiodine whole body scan.

**Table 2:** Characteristics of the case reports.

Cases	Age	Surgical Approach	TC Association/ Histology	RAI	Metastases	Adjuvant Therapies	MSO Histology	Localization
Case 1 [29]	67	bilateral salpingo-oophorectomy, total thyroidectomy	no	yes	latero-cervical, skeleton	no	FVPTC	right ovary
Case 2 [3]	50	bilateral salpingo-oophorectomy, total hysterectomy	no	no	no	no	Classic PTC	right ovary
Case 3 [25]	55	left thyroidectomy, thyroidectomy totalization, bilateral salpingo-oophorectomy	yes/FVPTC	yes	no	no	PTC with Hurtle cells subtype	right ovary
Case 4 [20]	33	hysterectomy bilateral salpingo-oophorectomy, peritoneal washing, total thyroidectomy, abdominal debulking	no	yes	intraperitoneal	no	FTC	right ovary
Case 5 [26]	48	left ovariectomy, total thyroidectomy	no	yes	intraperitoneal, liver	chemotherapy	PTC with columnar cells subtype	left ovary
Case 6 [22]	29	left ovariectomy, total thyroidectomy	no	no	no	no	Classic PTC	left ovary
Case 7 [24]	55	left ovariectomy, colonic nodule removal, total thyroidectomy	no	yes	intraperitoneal, liver, lung, spleen	no	FTC	left ovary
Case 8 [5]	36	total thyroidectomy, hepatic nodulectomy, citoreductive peritonectomy	no	yes	intraperitoneal, liver	no	FVPTC	right ovary
Case 9 [19]	49	hysterectomy, bilateral salpingo-oophorectomy, infracolic omentectomy, total thyroidectomy	no	yes	liver, peritoneal, pelvic	no	not mentioned	not mentioned
Case 10 [27]	50	salpingo-oophorectomy	no	no	no	no	FTC	left ovary
Case 11 [28]	30	left salpingo-oophorectomy, total thyroidectomy	no	yes	skeleton, lung	no	PTC with Hurthle cells subtype	left ovary
Case 12 [21]	41	right lower quadrant mass removal, total thyroidectomy	no	yes	intraperitoneal	no	FTC	right ovary
Case 13 [30]	11	left oophorectomy	no	no	no	no	Classic PTC	left ovary
Case 14 [23]	36	multiple pelvic mases removal, liver nodule removal, total thyroidectomy	no	yes	liver, intraperitoneal	retinoic acid	not mentioned	right ovary

TC—thyroid cancer; RAI—radioiodine therapy; MSO—malignant struma ovarii; FVPTC—follicular variant of papillary thyroid carcinoma; PTC—papillary thyroid carcinoma; FTC—follicular thyroid carcinoma.

## Data Availability

The authors confirm that the data supporting the findings of this study are available within the article.
